# Os peroneum imaging: normal appearance and pathological findings

**DOI:** 10.1007/s13244-016-0540-3

**Published:** 2017-01-05

**Authors:** Stefano Bianchi, Chandra Bortolotto, Ferdinando Draghi

**Affiliations:** 1 CIM SA, Cabinet Imagerie Médicale, Genève, Suisse; 2 Radiology Institute, University of Pavia, Pavia, Italy

**Keywords:** Os peroneum, Peroneus longus tendon, Painful os peroneum Syndrome, Ankle injuries, Diagnostic imaging

## Abstract

The os peroneum (OP) is a small sesamoid bone located inside the peroneus longus tendon (PLT), close to the cuboid. The OP can be the cause of pain and can be associated with lesions of the PLT. OP involvement in PLT disorders is frequently misdiagnosed by radiologists. Painful os peroneum syndrome (POPS) refers to a variety of conditions presenting with pain localized on the lateral aspect of the cuboid area. The syndrome can be observed as a consequence of local acute trauma such as ankle sprains or chronic overuse. Because of its intra-tendinous location, in tears of the peroneus longus tendon, the OP can show changes in its morphology or position, depending on the location of the tendon’s tear. Based on the level of the PLT tears, we propose a classification in three subtypes: tears localized proximal to the os peroneum (type I), at its level (type II) or distal to it (type III). These tears present with different changes on OP morphology or location. The aim of this article is to review the normal anatomy, imaging appearance and differential diagnosis of disorders of the OP as well as post-treatment imaging.

*Teaching points*

*• PLT tears can be classified in three subtypes according to OP location.*

*• POPS is characterized by pain on the lateral aspect of the cuboid.*

*• OP involvement in PLT disorders is frequently misdiagnosed by radiologists.*

## Introduction

Disorders of the peroneal tendons (PT) are frequent in the everyday practice of rheumatologists, orthopaedics, sports medicine doctors and physiotherapists [1–4]. The os peroneum (OP) is a small sesamoid bone located inside the PLT, close to the cuboid [5]. It is seen in 5–26% of the population [6] and is bilateral in 60% of patients [7]. OP involvement in PT pathologies is frequently misdiagnosed by radiologists [8, 9]. Nevertheless, the OP can be the cause of pain and can be associated with lesions of the PLT [10]. While in the majority of cases history and physical examination are able to guide towards a correct diagnosis, imaging is often necessary to confirm the clinical suspicion and choose between surgical and medical treatment. The aim of this article is to review the normal anatomy and imaging appearance of disorders of the OP.

## Normal anatomy of the OP (Fig. 1)

**Fig. 1:**
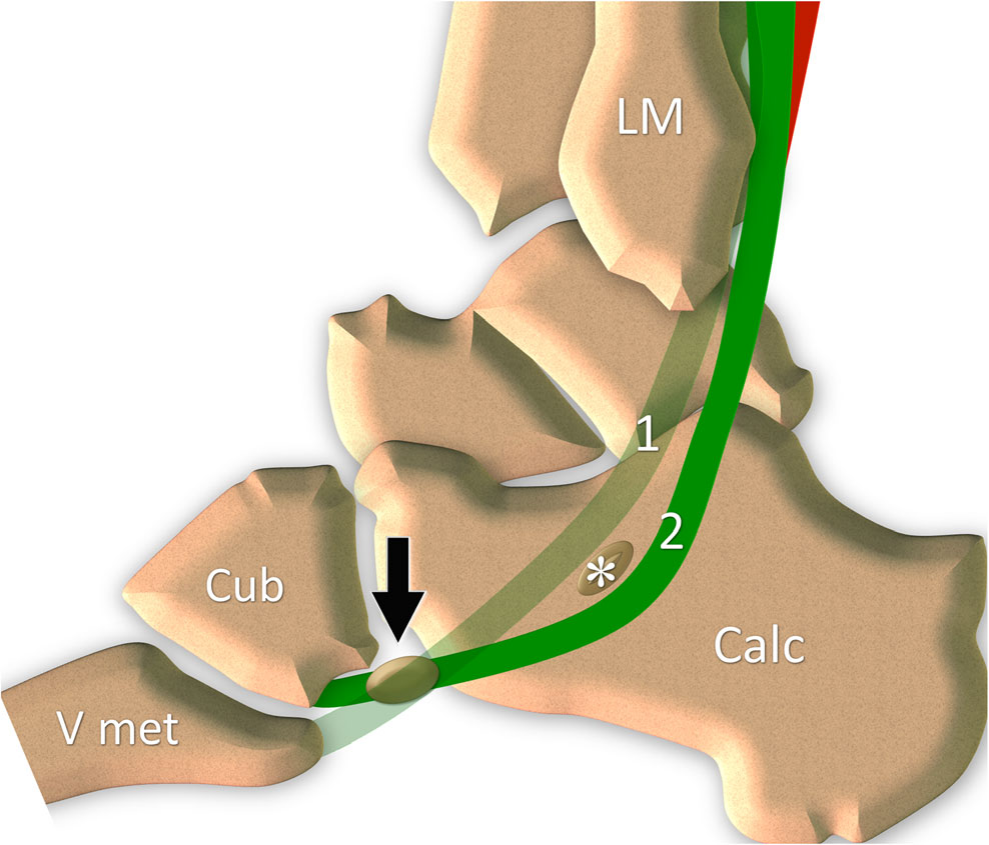
Os peroneum normal anatomy. Peroneal tendons (peroneus brevis 1, peroneus longus 2) travel along the lateral surface of the calcaneus that presents a small bony prominence, the peroneal tubercle (*asterisk*). The OP can be found inside the tendon at the level of the calcaneocuboid joint (*black arrow*). *LM* lateral malleolus, *Calc* calcaneus, *Cub* cuboid, *V met* fifth metacarpal bone

The PT complex is made up of the muscles and tendons of the PLT and peroneus brevis tendon (PBT), their common synovial sheath, the superior and inferior retinaculum, and the OP [5, 11–15].

The peroneal longus (PLM) and brevis (PBM) muscles are located inside the external compartment of the leg, lateral to the fibula. The PLM originates from the proximal portion of the lateral surface of the fibula and from the nearby intermuscular septum. The PBM originates from the distal third of the lateral surface of the fibula and from the nearby intermuscular septum. At the level of the leg’s median third, the PLM extends through a superficial, flat aponeurosis that covers the PBM. The PBM presents a myoaponeurotic junction distally located with regard to that of the PLM. Both muscles are innervated by the peroneal superficial nerve. The peroneal muscles are supplied by two principal source arteries, the anterior tibial artery and the peroneal artery.

At the malleolar region, the two tendons reflect against the posterior aspect of the lateral malleolus. At this level they run inside an osteofibrous tunnel (proximal tunnel) made up of the concave bony gutter and the thick fibrous superior retinaculum. This inserts into the lateral surface of the malleolus and guarantees the stability of the tendons during ankle movement, thus preventing their anterior luxation during movement of the ankle.

Distal to the lateral malleolus, the PT travel along the lateral surface of the calcaneus, which presents a small bony prominence, the peroneal tubercle (PTub). The peroneus brevis tendon (PBT) runs superiorly while the PLT runs inferiorly to it. At this level, the two tendons run inside another osteofibrous tunnel (distal tunnel), stabilized against the calcaneus thanks to the inferior retinaculum, which inserts on the apex of the PTub. The tubercle acts as a pulley for the PLT that reflects on its inferior surface.

Distal to the PTub, the PBT travels straight forward, to insert into the base of the fifth metatarsal bone.

The PLT, after reflecting against the cuboid, reaches the plantar region and attaches to the first and second metatarsal bones.

The two PT are enclosed by a common synovial sheath allowing optimal sliding of the tendons during movements of the ankle and/or contraction of the peroneal muscles. The sheath is made of a parietal layer adherent to the paratendinous soft tissue and a visceral layer adherent to the tendons. Proximal to the PTub the sheath divides to individually surround the two tendons. In some cases, the sheath surrounding the PLT is longer and extends from the cuboid tunnel to the plantar aspect of the foot. In physiologic conditions, it contains only a small amount of synovial fluid.

The OP is a small sesamoid bone which can be found inside the PLT at the level of the calcaneocuboid joint. The OP is ossified in around 20% of the population, and it is bilateral in around 60% of cases [7, 16, 17]. This small sesamoid bone can show different sizes and can also be bipartite or multipartite. A recent cadaveric study described the normal shape and size of the OP on 36 cadavers [18]. The ossicle presented a flattened oval shape, with one or two concave articular deep surfaces. The average thickness was 4 mm and length 13 mm. A bipartite bone is common and can be seen in around 30% of cases [19].

The presence of an OP or a bipartite OP can be clinically significant. The differential diagnosis between the ossicle and a cortical avulsion or a soft tissue calcification can be difficult in patients with pain in the lateral side of the foot. Similarly, a multipartite bone must be differentiated from a fractured one.

The origin of OP development is controversial. Some authors advocate an embryonic development of the ossicle [20], while others [18] believe that the OP develops from a “stress response”. A recent cadaveric study from Guimera and colleagues demonstrates that a precursor of the OP is already present during the foetal period [20]. Mittal et al. propose that development of the OP follows local stresses to the tendon, leading to its thickening and secondary ossification [18].

## Normal imaging of the OP

The OP can be visualized with several imaging techniques.

**Fig. 2:**
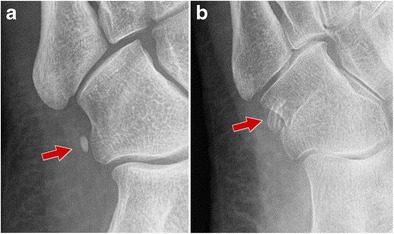
Normal os peroneum - standard radiograph. *2A* Single os peroneum (*arrow*) visualized as an oval, well-corticated ossicle located close to the calcaneocuboid joint. *2B* Bipartite os peroneum (*arrow*): two or more round fragments showing well-defined sclerotic margins

**Fig. 3:**
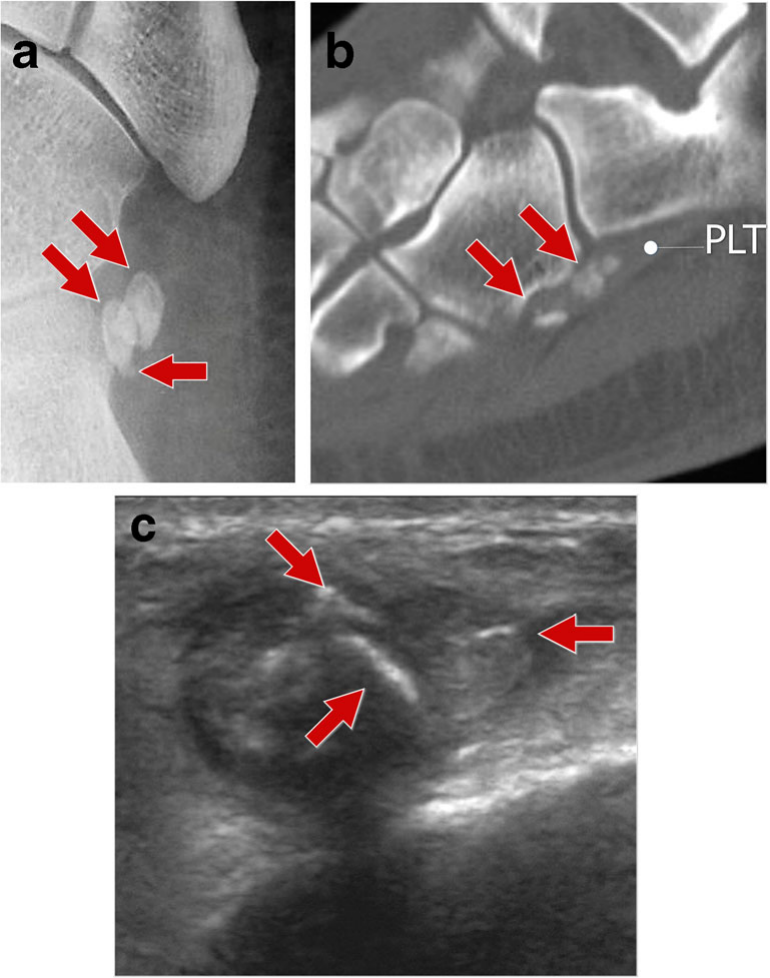
Multipartite os peroneum (*arrows*; different patients). *3A* Standard radiograph. *3B–C* Computed tomography and ultrasound. Accurate evaluation of the bone margins is of the utmost importance for differential diagnosis with a fracture. CT is the technique of choice to differentiate a multipartite OP from a non-displaced fracture. *PLT* peroneus longus tendon

Internal oblique radiographs of the foot can visualize the OP as an oval, well-corticated ossicle located close to the calcaneocuboid joint. The size, margins and location of the OP are easily assessed (Fig. 2A). In multipartite OP, two or more oval fragments showing well-defined sclerotic margins are noted (Figs. 2b and 3). Accurate evaluation of the bone margins is of the utmost importance for differential diagnosis with a fracture.

**Fig. 4:**
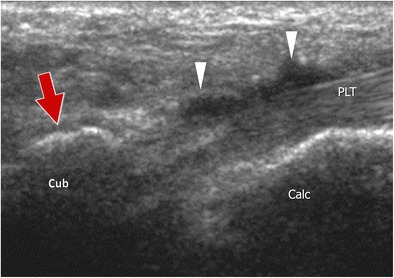
Normal os peroneum – ultrasound. The transducer is moved forward along the PLT until the cuboid (*cub*). A small amount of synovial fluid can be visualized along the PLT (*arrowhead*). When present, the OP is visualized at this level as a hyperechoic structure showing posterior shadowing (*arrow*)

Ultrasound (US) allows an analysis of the OP when using a high-frequency transducer and a rigorous examination technique (Fig. 4) [5, 21]. The examination starts at the PTub, which can be visualized in the oblique coronal plane as a hyperechoic eminence. Once the PTub is detected, the transducer is moved forward along the PLT to the cuboid. When present, the OP is visualized at this level as a hyperechoic structure showing posterior shadowing [22]. Once detected, the OP must be analysed on both axial and coronal oblique planes. Although US allows evaluation of the presence, multipartite appearance and size of the sesamoid, only the outer surface of the ossicle can be judged. The distal portion of the PLT can then be assessed in the plantar aspect of the foot. US is of value in guiding local interventional procedures such as corticosteroid injections in the peroneal tendon sheath, calcaneocuboid joint and local soft tissues [23].

**Fig. 5:**
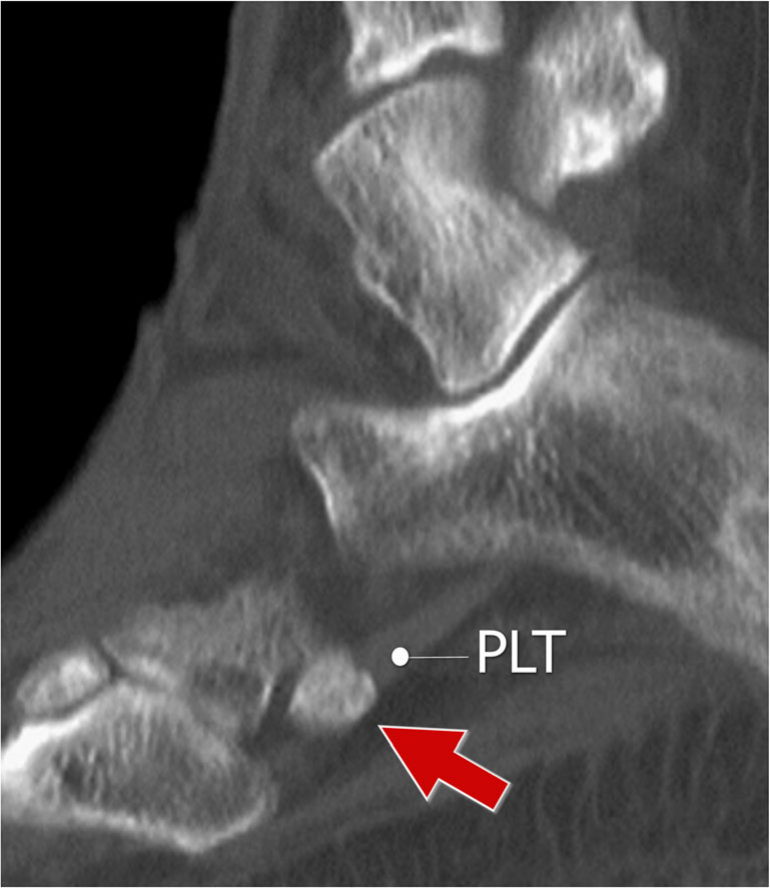
Normal os peroneum – computed tomography. Sagittal reconstruction along the PLT allows a precise evaluation of the OP (*arrow*)

Computed tomography (CT) allows a more precise evaluation of the OP, particularly when it is realized on a multidetector scanner with sub-millimetre collimation and oblique reconstruction on several planes (Fig. 5). It is the technique of choice for differentiating a multipartite OP from a non-displaced fracture.

**Fig. 6:**
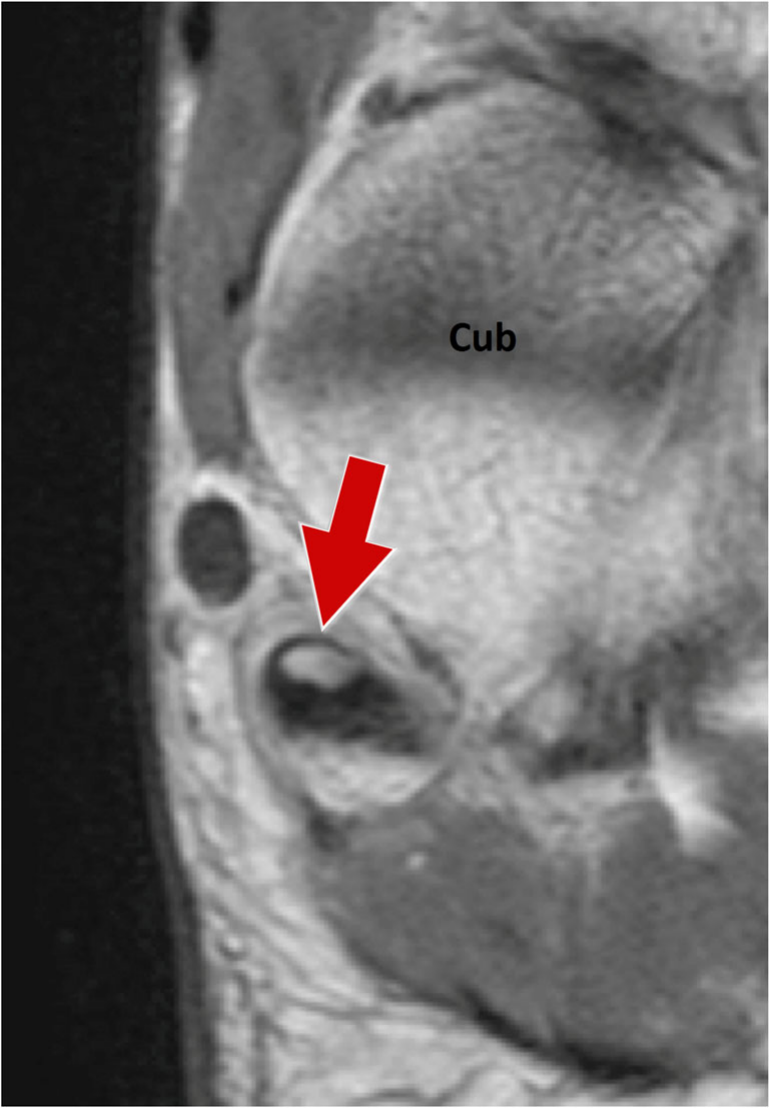
Normal os peroneum – magnetic resonance imaging. Coronal T1w plane. MRI allows optimal evaluation of the OP (*arrow*) bone marrow, which shows a signal similar to that of the adjacent cuboid and also correctly depicts pathologies of the synovial sheaths. *Cub* cuboid

Magnetic resonance imaging (MRI) allows an accurate evaluation of the OP (Fig. 6). The OP can be appreciated on axial, sagittal and coronal planes, especially if a 3D technique and small slice thickness are employed. Since the OP is located in a transition zone between the lateral and plantar course of the peroneus longus tendon, it is important to properly study this structure by orienting axial planes perpendicular to the long axis of the metatarsal shaft [22]. A correct orientation of planes helps minimize “magic angle” artefacts and also to correctly depict pathologies of the synovial sheaths. MRI allows optimal evaluation of the OP bone marrow, which shows a signal similar to that of the adjacent cuboid, hyperintense on T1w sequence and hypointense on T2w with fat suppression. Assessment of the cortex is difficult because of the hypointense signal of both the cortical bone and the tendon which encases it.

When no proper OP is visible, and only a fibrocartilaginous node is present, it is important to avoid one of the most frequent pitfalls of this situation. On MRI, the fibrocartilaginous node appears as a “pseudo tear”. Due to its chemical composition, it has an intermediate signal and can be mistaken as a real tear by an inexperienced radiologist [24].

Bone scintigraphy can detect the uptake in the area of the OP in chronic lateral foot pain with an unclear clinical history (micro-traumas or mismanaged non-recent traumas). In these cases, the nuclear medicine specialist must bear in mind the possibility that an uptake in this area can be related to a pathology of the OP [25].

## Imaging findings in pathologic conditions

### Painful os peroneum syndrome (POPS)

POPS refers to a variety of conditions presenting with pain localized on the lateral aspect of the cuboid area or on the OP [10].

**Fig. 7:**
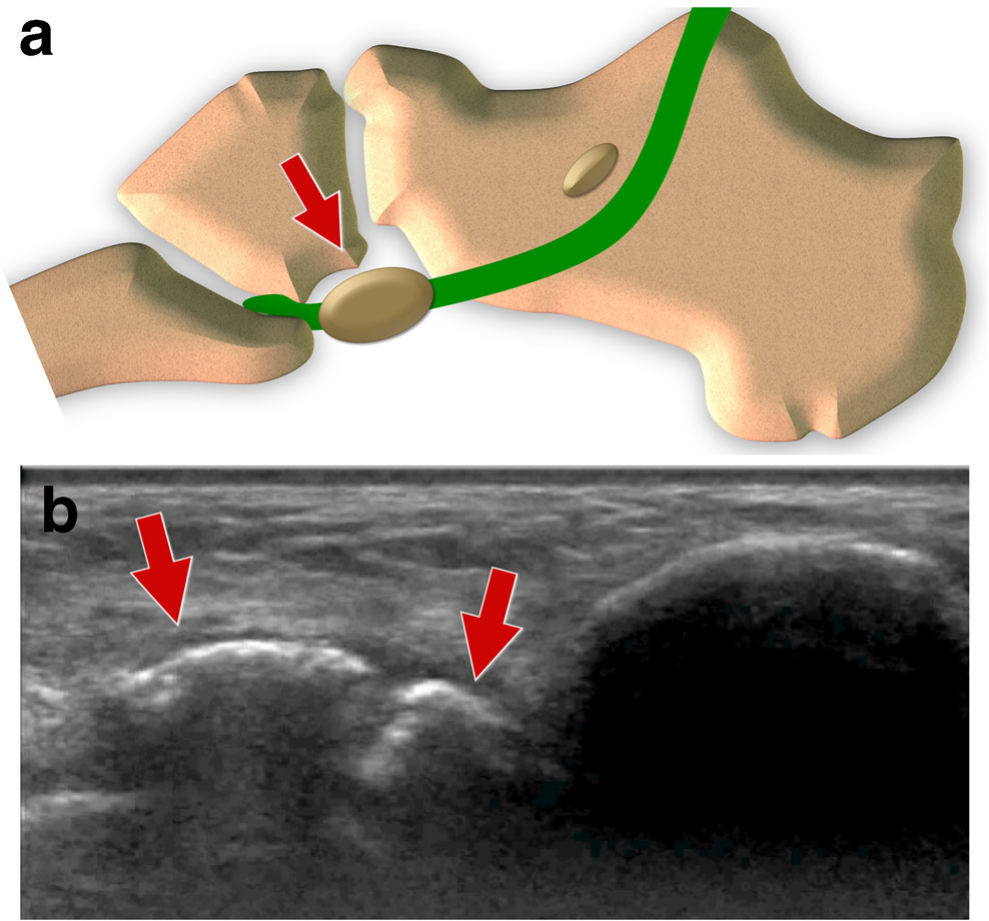
Hypertrophic os peroneum. *7A* Scheme *7B* Ultrasound. Hypertrophic OP facilitated a local impingement

The syndrome can be observed as a consequence of local acute trauma such as ankle sprains. In these cases, tears of the PLT or acute OP fractures are typically observed. Chronic overuse can also lead to POPS secondary to bone marrow oedema of the OP and cuboid, OP stress fractures, local impingement or partial tears of the PLT [10, 17, 26, 27]. As a consequence of the marked reflection of the PLT against the cuboid and secondary local stresses, it is not surprising that local friction between the two structures can lead to a local mechanical conflict [17, 28, 29]. Anatomic variation such as a hypertrophic OP, as well as overuse including cases of sport activities or hyper-supination of the ankle, can also facilitate a local impingement (Fig. 7) [10]. Patients present with well-localized pain over the lateral aspect of the tarsal region, usually exacerbated by walking, running and other physical activities.

**Fig. 8:**
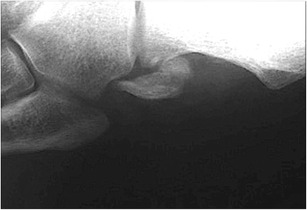
POPS – standard radiograph. Radiographs can show an enlarged and sclerotic OP suggestive of a stress fracture

In acute fractures, standard radiographs show multiple fragments of the OP, typically presenting sharp borders. This must be differentiated from a multipartite OP, in which the fragments are rounded and present regular and sclerotic cortical edges [30]. Correlation with clinical findings can aid in differential diagnosis. In chronic stresses, radiographs can show an enlarged and sclerotic OP suggestive of a stress fracture (Fig. 8).

In the majority of cases, US does not show abnormalities of the OP [28], which shows a regular and smooth cortical margin (Fig. 7b). Bone marrow oedema cannot be detected by US. Colour Doppler US can detect inflammatory hyperaemia of the adjacent soft tissues reflecting a local inflammation. These findings are detected only in the most severe cases and need an adequate examination technique as well as a high degree of suspicion by the examiner. When needed, US allows a real-time, accurate local therapeutic injection.

CT can assess the morphology of the OP well and allows a definite diagnosis of its acute or stress fractures, and in some cases points out erosions of the cuboid cortical bone [28]. This technique does not allow detection of bone marrow oedema or changes in the adjacent soft tissues.

**Fig. 9:**
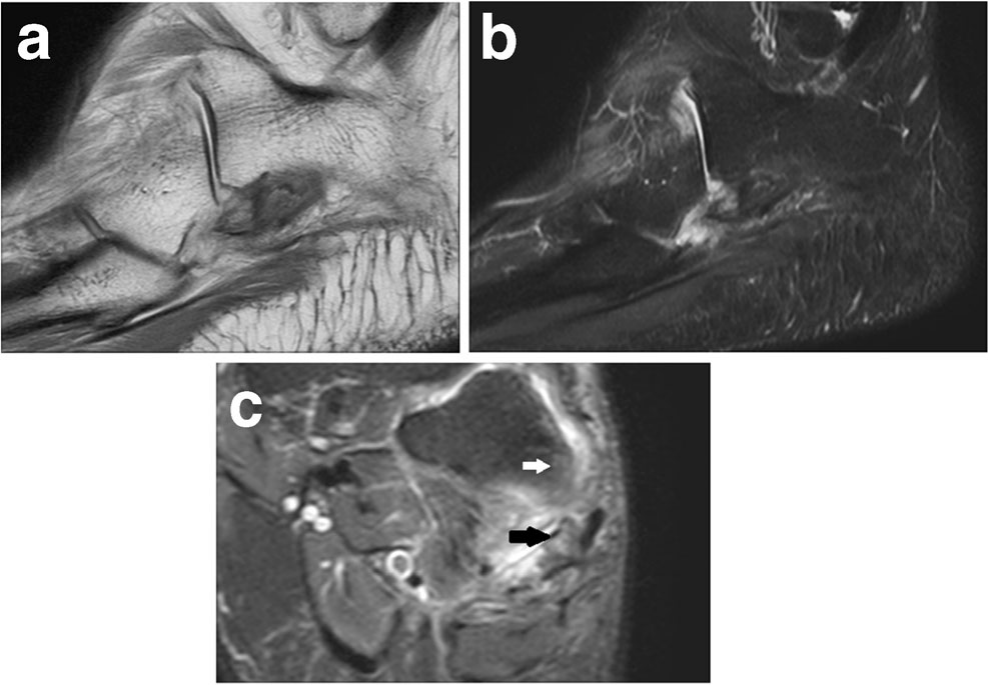
POPS – magnetic resonance imaging. *9A* T1w image, sagittal plane. *9B* T2w fat suppression image, sagittal plane. *9C* T2w fat suppression, coronal plane. MRI shows oedema of the bone marrow of the OP (*black arrow*) and cuboid (*white arrow*) as well as inflammatory changes in soft tissues

MRI is the gold standard in diagnosing POPS, since it shows pathologic changes of the bone marrow of the OP and cuboid as well as of soft tissues. Oedema appears hypointense on T1w sequence and hyperintense on T2w sequence with fat suppression (Fig. 9). After gadolinium administration, a local enhancement is detected. Nevertheless, MRI is inferior to CT in the assessment of the cortex of the OP.

**Fig. 10:**
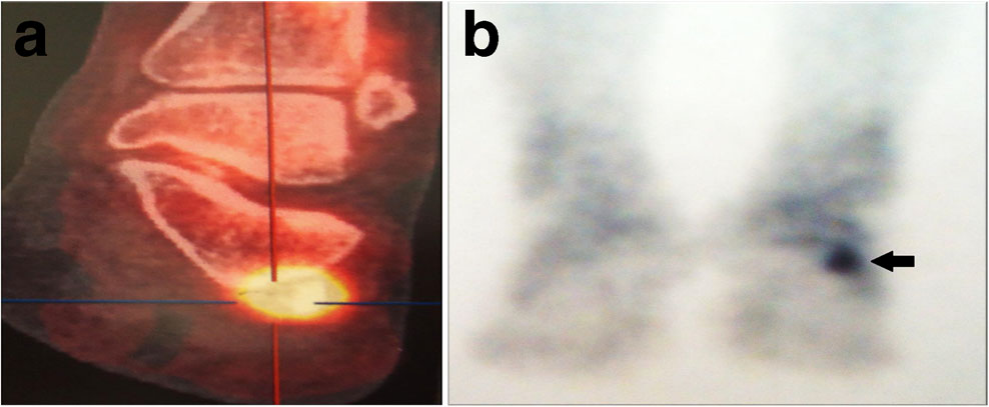
POPS – nuclear medicine. *10A* Positron emission tomography. *10B* Bone scintigraphy. Nuclear medicine can detect the uptake in the area of the OP (*arrow*) in chronic lateral foot pain

Bone scintigraphy can detect the uptake in the area of the OP in chronic lateral foot pain. In these cases, that uptake can be related to a pathology of the OP [25] (Fig. 10).

### OP in PLT tears

**Fig. 11:**
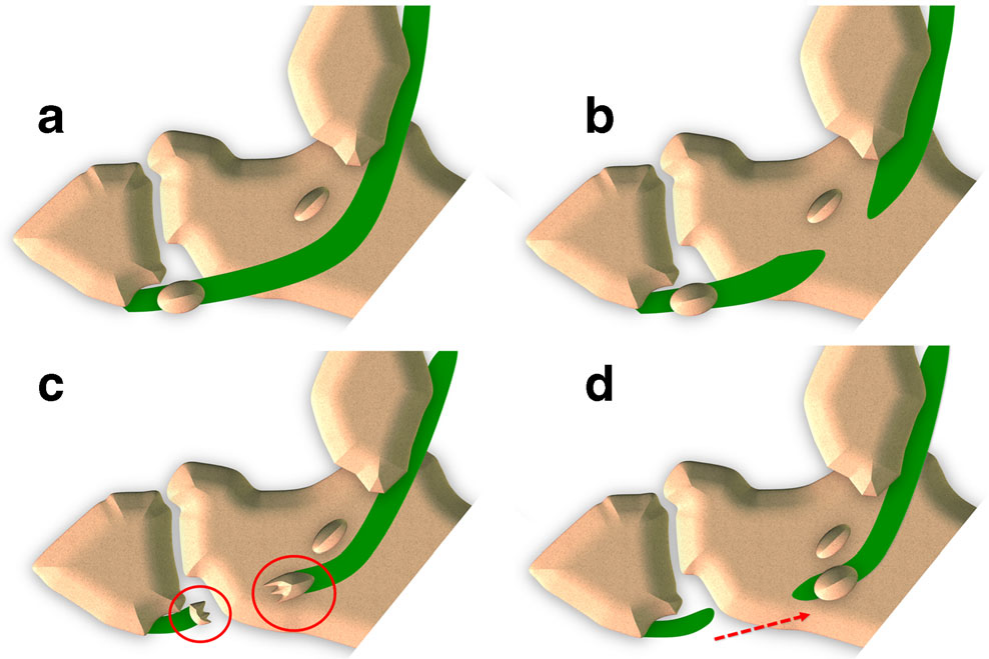
PLT tears – Scheme. *A* normal, *B* tear localized proximal to the OP (type I), *C* tear localized at OP level (type II), *D* tear localized distal to the OP (type III)

Because of its intra-tendinous location, in tears of the PLT, the OP shows changes in its morphology or position. Based on a review of the literature [1–4, 8–10, 15, 16, 23], as well as our practice experience, we propose a classification of tears of the PLT into three subtypes: tears localized proximal to the OP (type I), at its level (type II) or distal to it (type III) (Fig. 11).

#### Type I tears (Fig. 12)

**Fig. 12:**
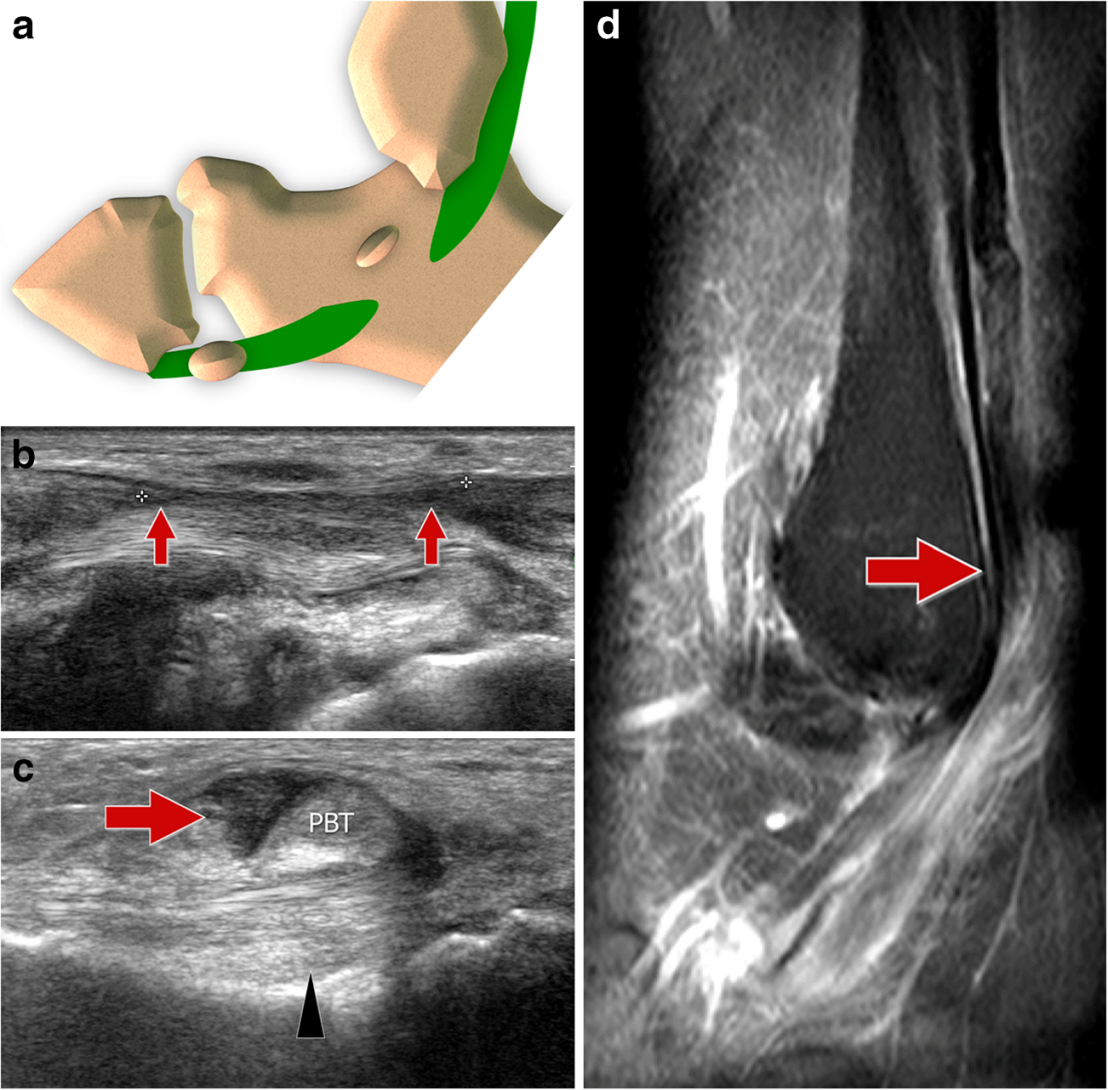
PLT tear type I. *12A* Scheme *12B* Ultrasound image. Longitudinal scan (tendon long axis). *Arrows* show the two tendon stumps and the gap between them. *12C* Ultrasound image. Transverse scan (tendon short axis). The peroneus longus tendon is not visible and is replaced by effusion (*arrow*). Peroneal calcaneal ligament (*arrowhead*). *PBT* peroneus brevis tendon. *12D* Magnetic resonance imaging. Retracted proximal stump of the peroneus longus tendon (*arrow*)

When the PLT tear is proximal to the OP, the ossicle presents a normal appearance and localization. This lesion cannot be diagnosed with radiographs or CT, which show a normal OP, but can be detected with ultrasound and MRI as irregularity of the tendons with local discontinuity usually associated with tenosynovitis.

#### Type II tears (Fig. 13)

**Fig. 13:**
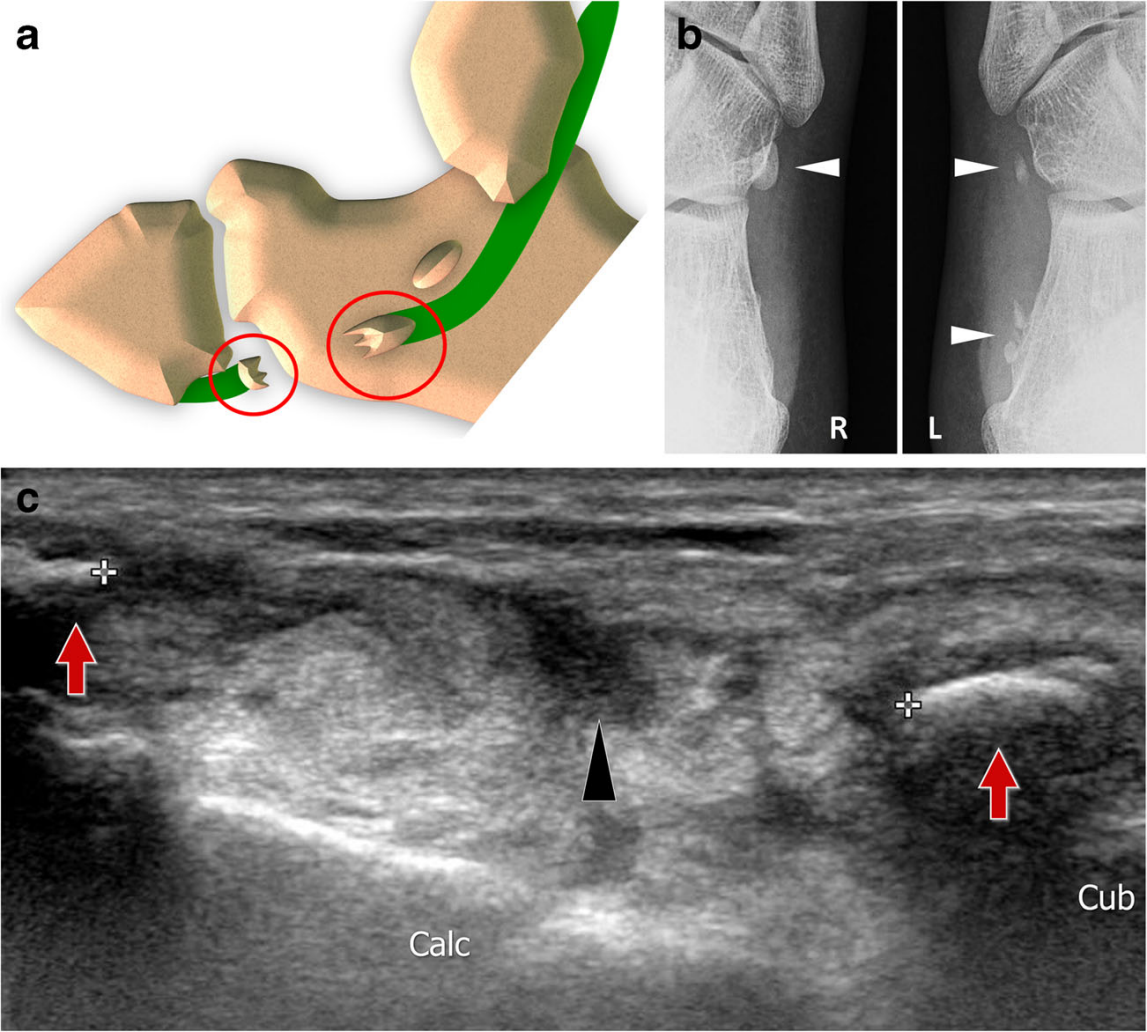
PLT tear type II. *13A* Scheme *13B* Standard internal oblique radiographs. On the left side, a lesion at the level of the OP with separation between fragments (*arrows*) of more than 6 mm. On the right side, the contralateral normal OP (*arrow*). *13C* Ultrasound. US clearly shows the two fragments (*arrows*) and the hematoma between them (*arrowhead*)

Tears of the PLT at the OP level present as ossicle fractures with two or more bone fragments of different sizes. As noted, OP fractures can be acute or chronic and present as a POPS syndrome. Diabetes mellitus is a factor facilitating fractures of the OP [31]. Whatever the pathologic mechanism, it implies an acute mechanical tension exerted by the PLT. Due to the persistent mechanical tension exerted by the PLT, a displacement of the fracture fragments can be readily evident or visualized only in the following days (delayed displacement).

Standard lateral and internal oblique radiographs are extremely useful for detecting an OP fracture [35], and show the posterior displacement of the proximal fragment that is usually larger (Fig. 13b). A separation between fragments of more than 6 mm or significantly increased displacement in subsequent control radiographs is associated with a complete lesion of the PLT [32, 33]. A progressive displacement, with radiographs obtained both immediately after the trauma and some days later showing a progressive increase in fragment distance, is an argument in favour of a complete tendon lesion [19, 32–34]. The proximal fragment is usually found anterior to the PTub of the calcaneus, inside the inferior osteofibrous tunnel. After strong contraction, the proximal fragment can be found posteriorly to the PTub, at the level of the posterior subtalar joint. When the fracture is not displaced, its irregular appearance and the absence of round sharp margins are the main aspects to take into account for a differential diagnosis between a fracture and a multipartite OP. In patients with a posterior displacement which is not readily evident, obtaining a contralateral radiograph can easily show even slight migration of the ossicle.

US can easily show a fracture of the OP when a significant displacement (>6 mm) of the posterior fragment is present. In these cases, US shows the bone fragments localized inside the tendon along the lateral side of the calcaneus. The retracted proximal fragment is usually found at the level of the PTub. The distal fragment can be more difficult to detect because of its smaller size. The distance between the two can be accurately measured (Fig. 13c). This can aid preoperative planning in order to correctly plan surgical incision points. In recent traumas, US can also show the hematoma between the two fragments and the associated inflammatory soft tissue phenomena. Colour Doppler can detect local hyperaemia. Local pressure exerted under US guidance is painful.

CT can detect the presence of the fracture even if minimally displaced, especially if performed with a multidetector scanner. Retraction of the proximal fragment is easily appreciated.

MRI is less useful than CT in analysing the bone cortex in non-displaced fractures, but allows the detection of bone marrow oedema and adjacent soft tissue inflammation in fat saturation images. In displaced fractures, MRI can easily detect the position of the retracted fragment and can demonstrate possible impingement of the retracted fragment at the level of the PTub inside the distal tunnel. MRI impingement features are similar to those of classic POPS but located more proximally. We call this lesion the “proximal POPS”. In addition, MRI shows tendon lesions and effusion inside the synovial sheath (seen as a hyper-signal on T2w sequence with fat suppression).

#### Type III tears (Fig. 14)

**Fig. 14:**
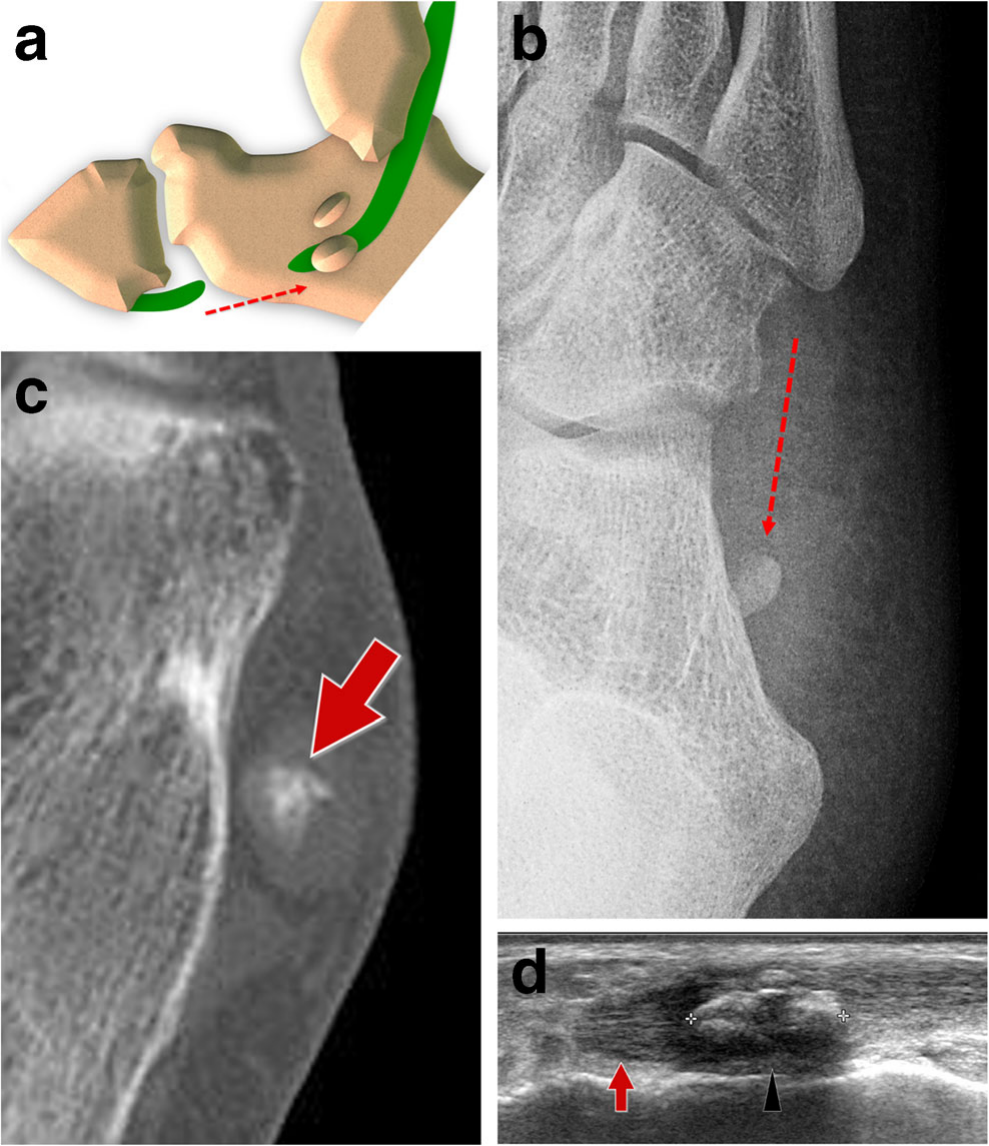
PLT tear type III standard retraction. *14A* Scheme *14B* Standard internal oblique radiograph. The OP shows a normal appearance but is posteriorly dislocated (generally less than 2 cm). *14C* Computed tomography. CT shows a regular appearance of the displaced sesamoid bone. *14D* Ultrasound. The degree of the dislocation and the size of the proximal tendinous stump (*arrows*) are easily measured. OP (*arrowhead*)

**Fig. 15:**
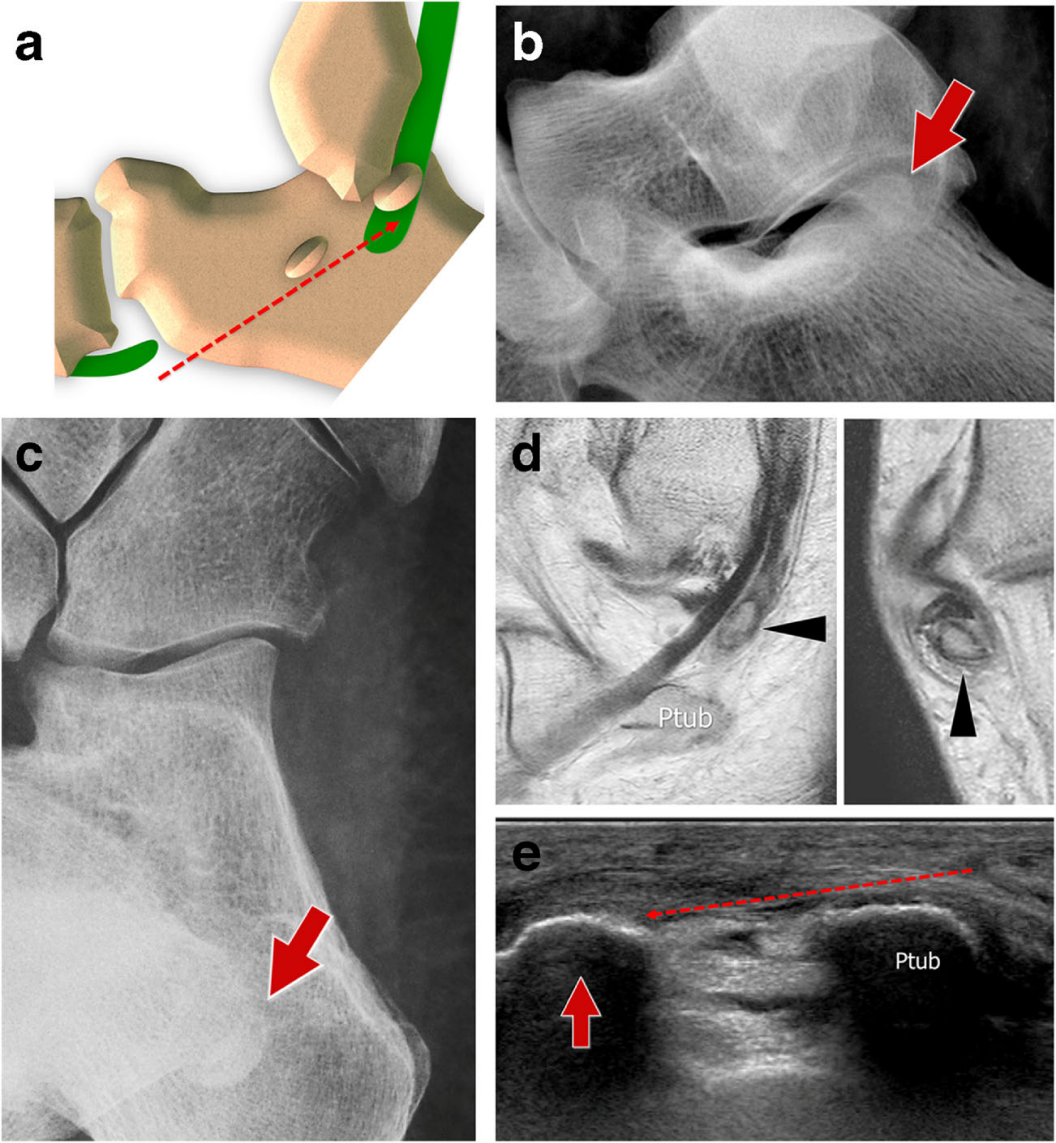
PLT tear type III proximal retraction. *15A* Scheme: proximal retraction after strong contraction. *15B–C* Lateral and internal oblique radiograph. The OP (*arrows*) can be found at the level of the posterior subtalar joint. *15D–E* Magnetic resonance imaging and ultrasound. The OP (*arrows*) can be found posteriorly to the PTub

When tears of the PLT are located distal to the OP, the sesamoid shows a normal appearance even though it is posteriorly displaced, because of the traction exerted by the peroneus longus [36, 37]. Displacement is generally less than 2 cm [38], as the sesamoid bone is stuck inside the distal osteofibrous tunnel at the level of the PTub. As already noted for type II tears, after strong contraction, the OP can be found posterior to the PTub, at the level of the posterior subtalar joint (Fig. 15). In this case, it can be misinterpreted as an os trigonum in an unusual position. This happens rarely, especially if the OP is small in size [33].

The internal oblique radiograph is the best way to highlight the posterior unusual localization of the OP. On lateral radiographs, this small bone is hard to localize because it is superimposed on the calcaneus. For these reasons, this diagnosis can be challenging for less experienced radiologists and is generally easier when a clinical suspicion is present. A comparison with previous radiographs is extremely important because it can confirm the displacement of the OP (with a normal localization in the initial radiograph) [19, 34, 39]. When previous radiographs are not available, a radiograph of the contralateral ankle can be obtained in order to show a typical localization of the contralateral OP if one is present.

US easily confirms type 3 tears. The degree of the displacement and the size of the proximal tendinous stump are easily measured. This can aid preoperative planning in order to correctly plan surgical incision points. Lastly, the alterations of the tendon sheaths are visible with US: effusion, sheath thickening and hyperaemia.

CT shows a regular appearance of the displaced sesamoid bone. The main differential diagnosis is with an accessory os subfibulare. Unfortunately, the tendon lesion is not evident in most cases.

This type of lesion can be identified with MRI, especially after contrast medium injection. Coronal images easily show the proximal stump, the displaced OP and the empty tendon sheaths distally located.

## Post-treatment imaging findings

Treatments for OP-related pathologies are extremely varied, ranging from non-operative treatments even in high-level athletes [40], to repair of the fractured ossicle or excision of both or just the proximal fragment in fractures [6]. Newer approaches include those based on tendoscopic intervention [41].

To our knowledge, no studies are available on postoperative imaging of the PLT and OP. Hypothetically, imaging can detect acute complications such as infection or re-rupture and late complications such as reflex sympathetic dystrophy syndrome (RSDS) or neuromas (e.g. sural nerve distal cutaneous branches travel on the lateral aspects of the ankle).

## Conclusions

The actual prevalence of the OP pathology is difficult to assess, but in our opinion, it is probably underestimated. Pathologic involvement of OP is frequently misdiagnosed by radiologists.

When an OP fracture is suspected, standard radiographs allow for a correct diagnosis only if carefully evaluated. In case of doubt, the contralateral ankle or previous radiographs must be used for comparison.

MRI is an extremely powerful tool but needs proper plane and field of view selection. It is the gold standard for the detection of POPS.

When a PLT tear is suspected, US can be used as a first approach. Also for US, contralateral ankle evaluation is extremely useful.
